# Research literacy and its predictors among university students and graduates identified by machine learning and spatial analysis

**DOI:** 10.1038/s41598-025-19488-4

**Published:** 2025-10-13

**Authors:** Mohammed A. Mamun, Md. Abu Huraira, Momotaj Begum, Md. Hasibul Islam Jitu, Naoroj Muntashir, Md. Maruf Khan, Pronab Das, Sadikur Rahman, Umme Zaida Misma, Sajib Nath, Tamim Ikram, Rubiya Wazed, Marjia Khan Trisha, Md. Shabbir Ahamed, Md. Omar Faruk, Arpita Howlader Tisa, Aysha Siddiky, Sree Siddarth Shankar Devnath Satu, Abdul Kaium, Md. Shakibul Hasan, Sabrina Aktar, Md. Zahidul Hasan, Md. Mehedi Hasan, Mohammad Kibria, Tasnim B.K Chowdhury, Milan Kumar Das, Md. Abdulla Hell Kafi Patowary, Md. Hamed Hasan, Sharmin Akter, Anonna Haque, Kulsuma Bahar Bethi, Jannatul Ferdaus, Pintu Chandra Shil, Md. Emran Hasan, Moneerah Mohammad ALmerab, Firoj Al-Mamun, Nitai Roy, David Gozal

**Affiliations:** 1 CHINTA Research Bangladesh, Savar, 1342 Dhaka Bangladesh; 2 Department of Public Health and Informatics, Jahangirnagar University, Dhaka, 1342 Bangladesh; 3 Department of Public Health, University of South Asia, Dhaka, 1348 Bangladesh; 4 Department of Public Administration, Jahangirnagar University, Dhaka, 1342 Bangladesh; 5 Department of Sociology, Noakhali Science and Technology University, Noakhali, 3814 Bangladesh; 6 Institute of Statistical Research and Training, University of Dhaka, Dhaka, 1000 Bangladesh; 7 Department of Chemical Engineering, Bangladesh University of Engineering and Technology, Dhaka, 1000 Bangladesh; 8 Department of Biochemistry and Molecular Biology, Primeasia University, Dhaka, 1213 Bangladesh; 9 One Health Institute, Chattogram Veterinary and Animal Sciences University, Chattogram, 4225 Bangladesh; 10 Department of Philosophy, Jahangirnagar University, Dhaka, 1342 Bangladesh; 11 Department of Public Health, Premier University, Chattogram, 4317 Bangladesh; 12 Department of Food Technology and Nutrition Science, Noakhali Science and Technology University, Noakhali, 3814 Bangladesh; 13 Department of Food and Nutrition, Govt. College of Applied Human Science, University of Dhaka, Dhaka, 1000 Bangladesh; 14 Saic College of Medical Science and Technology, Dhaka, 1216 Bangladesh; 15 Faculty of Nutrition and Food Science, Patuakhali Science and Technology University, Patuakhali, 8602 Bangladesh; 16 School of Science and Technology, Bangladesh Open University, Gazipur, 1705 Bangladesh; 17 Department of Acupuncture and Moxibustion & Tuina, Tianjin University of Traditional Chinese Medicine, Tianjin, 301617 China; 18 Department of Library and Information Science, National University, Gazipur, 1704 Bangladesh; 19 Department of Chemical and Biomedical Engineering, University of Missouri, Columbia, 65211 USA; 20 Department of Anthropology, Rajshahi University, Rajshahi, 6205 Bangladesh; 21 Department of Sociology, Shahjalal University of Science and Technology, Sylhet, 3114 Bangladesh; 22 Department of Biochemistry and Molecular Biology, Jahangirnagar University, Dhaka, 1342 Bangladesh; 23 Department of Biochemistry & Molecular Biology, Gopalganj Science and Technology University, Gopalganj, 8100 Bangladesh; 24 Department of Food Technology and Nutritional Science, Mawlana Bhashani Science and Technology University, Tangail, 1902 Bangladesh; 25 Bangabandhu Sheikh Mujib Medical College, Faridpur, 7800 Bangladesh; 26 Institute of Nutrition and Food Science, University of Dhaka, Dhaka, 1000 Bangladesh; 27 Department of Public Health, Hamdard University Bangladesh, Gazaria, 1510 Bangladesh; 28 Department of Public Health, North South University, Dhaka, 1229 Bangladesh; 29 Software College, Northeastern University, Shenyang, 110169 China; 30 Department of Psychology, College of Education and Human Development, Princess Nourah bint Abdulrahman University, Riyadh, 11671 Saudi Arabia; 31 Department of Biochemistry and Food Analysis, Patuakhali Science and Technology University, Patuakhali, 8602 Bangladesh; 32 Departments of Pediatrics, Biomedical Sciences, and Office of the Dean, Joan C. Edwards School of Medicine, Marshall University, West, VA 25701 USA; 33 School of Computer Science and Engineering, Nanjing University of Science and Technology, Nanjing 210094, China

**Keywords:** Research literacy, Predatory journals, Research training, Thesis students, Plagiarism, Research evaluation, Computational biology and bioinformatics, Medical research, Risk factors

## Abstract

The landscape of academic publishing has evolved dramatically, leading to a surge in publications and journals. The ‘*publish or perish*’ culture has resulted in undesirable practices, such as many researchers publishing in predatory journals due to institutional pressures and lack of awareness. While numerous studies have investigated knowledge of predatory journals, overall research literacy has remained underexplored. This study is the first to assess research literacy comprehensively, incorporating GIS and machine learning techniques alongside traditional statistical analyses. This study utilized a cross-sectional survey method with a questionnaire collecting information on socio-demographics, academic information, research training and experience, and research literacy. Traditional statistical analyses were performed using SPSS, while machine learning models were developed with Python and Google Colab. Supervised classification algorithms and mapping with R statistical software’s ‘bangladesh’ package. The findings revealed that over half of the participants had poor research literacy. Significant predictors of higher research literacy included satisfaction with research courses at university education, research course taken outside university , and research-related professional engagement. Machine learning analysis identified that taking research courses outside of university was the most impactful factor for research literacy, while researchers within family members had minimal influence. The Random Forest and CatBoost models performed strongly in predicting literacy, achieving accuracy rates of 73.04% and 71.57%, respectively, and precision values of 73.29% and 71.69%, respectively, with low log loss values of 0.57 and 0.56. GIS-based spatial analyses revealed regional disparities in research literacy (χ²=9.234, *p* = 0.236), with certain divisions exhibiting a higher prevalence of lower literacy. This study highlights that a substantial portion of the participants lack research literacy, which is associated with multiple factors. The findings suggest the need for intervention programs to enhance research practices and awareness among students and professionals, fostering a culture of academic excellence.

**Supplementary Information:**

The online version contains supplementary material available at 10.1038/s41598-025-19488-4.

## Introduction

Academic publishing has undergone significant changes over time, with notable increases in both the number of publications and journals. Fire and Guestrin^1^ observed a shift in publication patterns, remarking that researchers who started their careers in the 1970s typically published around 2 conference papers and 1.65 journal papers within ten years, and that such publication rates had increased to approximately 4 conference papers and 2.59 journal papers for those beginning their careers in the 2000s. In addition, the average number of publications in journals rose from 74.2 in 1999 to 99.6 in 2016. The rise of mega journals, characterized by their rapid peer-review processes and broad scopes, has also contributed to these trends. For example, PLOS One published over 20,000 articles in a single year^2^. In 2016 alone, about 200 journals published more than a thousand papers each^1^. Despite these changes, there remains a critical gap in our understanding of how well-prepared university students and graduates are to navigate legitimate and predatory publishing, a gap with serious implications for academic integrity worldwide.

The criteria for evaluating academic performance have undergone significant evolution, with metrics such as publications, citations, and a variety of impact factors now serving as indicators of both author performance and institutional preeminence^1^. Many academic institutions require faculty to meet specific publication quotas to maintain their rankings. In Turkey, for instance, publications in internationally reputed journals included in bibliometric services (e.g., Web of Science, Scopus, PubMed, etc.) are necessary for academic promotion^3^. However, this pressure can lead to undesirable practices, as evidenced by a study showing that a significant portion of those publishing in predatory journals felt compelled to pursue this avenue, with 41% and 25.3% needing to meet specific article quotas for tenure and promotion due to institutional mandates^4^. Furthermore, new hires in academia are frequently chosen based on their publication track records. Publications also play a decisive role in admission decisions to postgraduate and clinical residency programs, favoring candidates with a strong publication history due to their perceived ability to contribute to research output and secure funding^5^. PhD candidates are also typically required to publish in indexed journals to complete their degrees^3^. This emphasis on publishing, encapsulated by the term ‘*publish or perish*’, coined in the 1930s^6,7^, has created a culture where researchers may rely on solutions involving predatory journals out of fear of job insecurity. Initiatives lacking proper regulations, such as incentivizing publication without scrutiny of journal quality, further exacerbate this issue^3,8^. A study among Saudi Arabian nurse researchers revealed that 70% had experience publishing in predatory journals and that 65.7% were unaware of their predatory nature. Pressure to publish (22.9%), lack of confidence in publishing in high-quality journals (5.7%), inability to publish elsewhere (5.7%), and the allure of rapid publication (14.3%) were other important factors leading to predatory journal options^9^, and these findings are reflected in other studies^4,5^. Al Ryalat et al.^10^ noted that young researchers, particularly neurosurgeons, are often unaware of predatory journals and may prioritize quick publication without considering the significance of journal selection in the publication process. Thus, combination of pressure to publish and lack of awareness can easily lead early-career researchers into the trap of predatory publishing^11^.

Predatory journals, often listed on Beall’s List, are characterized by compromised or non-existent peer-review processes, lack of indexing services, and by universally claiming publication fees^12^. Many researchers fall prey to predatory journals due to inadequate literacy and understanding of legitimate journal criteria. A significant number of researchers reported having no prior knowledge of predatory journals (68.6%) and no training on the subject (75.7%)^9^. Medical students in Saudi Arabia also felt pressured to publish, with minimal guidance on how to avoid falling prey to predatory journals^13^. Proper mentorship from experienced colleagues can help young researchers navigate these pitfalls^14^, and familiarity with resources like Beall’s List and Directory of Open Access Journals (DOAJ) has been associated with improved recognition of predatory journals^15,16^. Educational interventions, such as a 1-minute infographic presentation, have shown remarkable efficacy in raising awareness to predatory publishing practices (7–97.5%)^10^. Therefore, enhancing research literacy is crucial to equipping individuals the knowledge and skills to make informed decisions regarding scholarly publishing. However, despite these global trends and mounting publication pressures, there is a critical lack of research systematically measuring research literacy and identifying its determinants—particularly in low- and middle-income countries. This study addresses two critical gaps: first, by developing and applying a comprehensive operationalization of research literacy; and second, by employing advanced analytical methods, including machine learning and Geographic Information Systems, to identify key predictors and regional variations. This study specifically addresses this gap by operationalizing and measuring research literacy as a multidimensional construct, and by identifying the predictors of research literacy among university students and graduates.

In this study, we define '*research literacy*' as a multidimensional construct that encompasses familiarity with key domains of academic publishing. We assess knowledge across ten core components: the peer-review process, predatory journals, open-access publishing, indexing databases (such as Scopus, PubMed, and Web of Science), citation metrics (including h-index and i10-index), impact factors and CiteScore, the Directory of Open Access Journals (DOAJ), Beall’s List, plagiarism and research misconduct, and preprints. These elements were measured through a structured questionnaire developed for this research. Existing studies have highlighted the critical importance of these domains. For instance, understanding the peer-review process is fundamental to maintain the quality and integrity of scholarly communication^17^. Awareness of predatory journals helps researchers avoid unethical publication venues that lack rigorous review and may exploit authors^11,18^. Knowledge of open-access publishing expands the reach and accessibility of research findings to a broader audience^19^. Familiarity with indexing databases and citation metrics allows scholars to identify reputable publication outlets and objectively assess research impact. Journal-level metrics, such as impact factors and CiteScore, further inform strategic decisions on manuscript submission^20,21^. Tools like the DOAJ and Beall’s List are valuable for distinguishing credible open-access journals from potentially predatory ones^12^, while an understanding of plagiarism and research misconduct is vital for upholding ethical standards in science^22^. Finally, awareness of preprints supports the rapid dissemination of new findings and encourages timely scientific dialogue^23^. Together, these components of research literacy equip researchers to navigate the complexities of academic publishing and uphold the standards of scientific inquiry.

While previous studies have extensively investigated knowledge, attitudes, and practices concerning predatory journals across various groups; for instance, nurse researchers^9^, orthopedic and trauma physicians^15^, dermatologists^14^, biomedical researchers^10^, medical students^13^, veterinary and medical authors attending scientific writing workshops^18^; no prior research has comprehensively assessed the full spectrum of research literacy outlined above. Moreover, little is known about regional variations in research literacy or the predictive factors associated with its levels. To address these critical gaps, this study systematically evaluates overall research literacy levels and examines regional disparities and predictors using advanced analytical methods. Specifically, we incorporated Geographic Information Systems (GIS) analysis, which uniquely enables visualization and identification of regional patterns and inequities in research literacy that might otherwise remain obscured in aggregated analyses^24^. Besides, we employed machine learning (ML) approaches due to their superior capacity to detect complex, non-linear interactions among predictors that traditional statistical models might overlook^25^. By integrating GIS and ML methods, this study provides a robust and nuanced understanding of research literacy, offering a significant methodological advancement and practical insights for improving research education and mitigating the risks associated with predatory publishing.

Although the present research is grounded in the context of Bangladesh, its findings hold broad international relevance. Many higher education systems around the world—especially those in rapidly developing and resource-constrained settings—face similar challenges in safeguarding academic integrity, improving research capacity, and protecting early-career scholars from exploitative publishing practices. By systematically examining research literacy in this context, our study offers insights and strategies that may inform global efforts to enhance scholarly publishing standards and research training across diverse academic environments. Given the lack of prior research on research literacy as a multidimensional construct, we intentionally included a wide range of demographic, academic, and research experience variables—such as, gender and age. This approach was informed by mixed and sometimes contradictory findings in the literature regarding the influence of these factors^5,14,16,26^, enabling both replication and exploration of potentially novel associations. For example, while some studies report higher research literacy or related knowledge among males, others find that gender differences are minimal or context-dependent^14,26^. Similarly, while age and experience often predict higher research-related knowledge, findings are not always consistent across populations^5,14,16^. As there has been no comprehensive assessment of research literacy incorporating these domains—especially in low- and middle-income countries—it was critical to examine these predictors in our Bangladeshi sample to identify context-specific patterns.

Therefore, the present study aims to: (1) comprehensively assess the level of research literacy among university students and graduates in Bangladesh; (2) identify key demographic, academic, and experiential predictors of research literacy using both traditional and machine learning approaches; and (3) explore regional variations in research literacy utilizing GIS. By addressing these aims, this study seeks to provide actionable insights for educators, policymakers, and institutions working to strengthen research capacity and academic integrity both within Bangladesh and in comparable international contexts.

## Methods

### Study design and participants

This cross-sectional study aimed to assess research literacy levels among current university students and recent graduates in Bangladesh. Eligible participants included individuals who had completed or were currently enrolled in bachelor or master’s degree programs, ensuring they had some academic research exposure. A non-probability sampling technique, combining convenience and snowball sampling, was utilized to recruit a heterogeneous group of participants from diverse academic backgrounds and universities. While this approach maximized reach, it may limit generalizability to the wider Bangladeshi university population, which is acknowledged as a study limitation. Data were collected via an online questionnaire distributed through Google Forms.

### Sample size calculation

The minimum required sample size was determined using the standard formula for cross-sectional surveys measuring proportions: $\:n=\frac{{Z}^{2}P(1-P)}{{d}^{2}}$, where Z = 1.96 (95% confidence), *P* = 0.5 (assuming maximum variability), and d = 0.05 (margin of error). This calculation yielded a minimum sample size of 384 participants. The survey collected 522 valid responses, but after cleaning the data to adhere to eligibility criteria (e.g., ensuring participants were Bangladeshi students or graduates) and removing inconsistent or incomplete responses, 508 responses were used for the final analysis. This sample exceeds the minimum required for prevalence estimation, and is comparable to those used in similar research on research literacy and related domains. Given the exploratory nature of this study and the diversity of participant backgrounds, the sample is considered adequate for the intended statistical and machine learning analyses.

### Questionnaire development

A comprehensive literature review informed the development of the questionnaire, which was piloted with about 20 students between February 19 and March 6, 2024. Expert review was used to strengthen content validity, and the instrument was iteratively revised based on feedback to improve clarity, face validity, and relevance. The online survey, which took approximately fifteen minutes to complete, consisted mainly of closed-ended questions and was divided into six sections: (i) socio-demographic variables, (ii) academic information, (iii) research training-related information, (iv) research publication-related information, (v) career-related information, and (vi) factors related to research literacy. The detailed questionnaire is provided in the supplementary file.

The initial questionnaire to assess research literacy was developed based on a comprehensive literature review and existing frameworks related to research literacy and scholarly publishing. To ensure content validity, the draft instrument was reviewed by a panel of three experts in research methodology, academic publishing, and medical education. Their feedback was used to refine and clarify items for relevance and coverage. The questionnaire was then modified to improve clarity and feasibility. While the scale demonstrated high internal consistency (Cronbach’s alpha = 0.939), we acknowledge that formal construct validation (e.g., factor analysis) was not conducted due to the exploratory nature of this study. Although a high Cronbach’s alpha indicates excellent internal consistency, it may also suggest potential item redundancy. During questionnaire development, we carefully reviewed item content to minimize overlap, but further psychometric assessment (such as inter-item correlation analysis or factor analysis) is recommended in future research to fully address this issue. We recommend future studies further assess construct validity.

### Data collection process

Data collection was conducted using a Google Form. The formal data collection took place from March 6 to 12, 2024. The Google Form was disseminated through various online platforms, including emails, messages, and posts on the *CHINTA Research* *Bangladesh’s* Facebook page and group, as well as across multiple educational groups. In addition, the participants and researchers involved in the project shared the questionnaire on their social media profiles. CHINA Research Bangladesh also organized a free webinar on ‘Research and Higher Study’, where webinar participants were invited to take part in the survey. Recruitment efforts aimed to maximize inclusion and coverage across disciplines and regions. At the beginning of the questionnaire, participants were provided with clear information about the survey’s objectives, as well as details regarding the ethics and rights of participants. Informed consent was obtained before proceeding with the study. Participation was voluntary and anonymous, and no incentives were offered, which may help reduce social desirability and response bias, although such biases remain possible in any online self-report survey.

### Measures

The selection of demographic, academic, and research training variables (such as gender, age, educational background, and research engagement) was informed by prior research on related domains—including studies of predatory publishing, academic publishing practices, and information literacy—which have shown mixed or context-dependent associations with these factors^5,14,16,26^. Given the lack of previous studies specifically on research literacy, an inclusive variable selection was adopted to both explore established predictors in a new context and to identify novel patterns within the Bangladeshi student and graduate population. This approach allows for comprehensive analysis and for generating findings that may inform future research and interventions.

#### Sociodemographic factors

The sociodemographic information collected encompassed multiple variables, including gender, age, marital status, permanent residence (rural or urban), current living place, sources of financial support, socioeconomic status based on family income, personal income level, research support within the family, and family members involved in research and their relationship to the participant. Participants’ marital status was categorized into three groups: married, unmarried/single, and in a relationship, with divorced status merged with unmarried/single due to the low number of responses. Participants’ districts were categorized into eight divisions. Socioeconomic status and personal income levels were classified into four groups: lower class (income less than 20,000), middle class (income between 21,000 and 40,000), high class (income between 41,000 and 60,000), and upper high class (income more than 61,000). In terms of family members engaged in research, the ‘others’ category included relationships such as uncle, cousin, brother-in-law, sister-in-law, and father-in-law.

#### Academic information

A wide-ranging academic information was collected from respondents, including their institute, field of study, and admission sessions for both bachelor’s and master’s programs. Bachelor’s program institutions were categorized into four groups: General (General and National), Private, Specialized (Technology, Engineering, and Agriculture), and Health Sciences (Medical, Nursing, Health Sciences, and Public Health). Fields of bachelor’s studies were broadly categorized into Life Sciences, Health Sciences, Social Sciences and Arts, Business, and others. For master’s studies, the Sciences category was included, encompassing agriculture, food, life science, and basic science. Additional information on academic sessions, session jams, and results was collected, noting that some students reported their most recent published results if still in education.

#### Research course-related information

Participants were asked if they had taken any research-related courses during their university education (both bachelor’s and master’s programs) or participated in research courses or training programs offered by external organizations. Information on the number of research courses taken outside university education was also collected. The effectiveness of these research courses, both inside and outside the university, was evaluated using a four-point Likert scale (not at all, somewhat, moderately, highly), with participants rating how beneficial these courses were for working on their thesis or publications.

#### Research publication and career-related information

This section included four questions. First, the status of the participants’ thesis was collected using categories: completed thesis, continuing thesis, unwillingness to do a thesis, undecided about doing a thesis, and no opportunity to do a thesis. Thesis status was then categorized into two groups: (i) thesis group (completed thesis and continuing thesis) and (ii) non-thesis group (unwillingness to do a thesis, undecided, and no opportunity). Information on participants’ involvement in any research projects beyond their thesis, their status (either continuing work or seeking publication), and the number of publications was also gathered. Additionally, participants were asked about their involvement in research-related jobs, their interest in building research-related careers, and their reasons for choosing research as a career, with a multiple-response question.

#### Research literacy

To assess research literacy, the study included ten questions aimed at measuring participants’ understanding of various aspects of scholarly publishing and research. These aspects included: (i) peer-review process, (ii) recognition of predatory journals, (iii) awareness of open-access publishing, (iv) familiarity with indexing sites, (v) familiarity with citation metrics, (vi) recognition of impact factors or site scores, (vii) knowledge of directories of open access journals, (viii) awareness of Beall’s List, (ix) awareness of plagiarism, and (x) familiarity with preprints. Items were developed based on literature review and refined through expert feedback and pilot testing to enhance clarity and relevance. Responses were on a 4-point Likert scale (0 = not familiar at all, 1 = slightly familiar, 2 = moderately familiar, and 3 = very familiar), with total scores ranging from 0 to 30. Participants’ scores were totaled, and based on the median score as a cutoff point (10), their research literacy was categorized into two groups: higher knowledge and lower knowledge. Cronbach’s alpha for the present study was 0.939, which was excellent.

### Ethical considerations

Before the study implementation, it received approval from the Institutional Review Board at Patuakhali Science and Technology University, Bangladesh [Reference Number: PSTU/IEC/2023/81]. Besides, the study adhered to the principles outlined in the revised Helsinki Declaration of 2013, ensuring ethical standards for human subject participation were met. Participants were informed about the purpose and objectives of the study through a brief description at the beginning of the questionnaire, where their written informed consent was sought for participation. They were assured of their right to withdraw from the study at any time without any obligation. It is important to note that no monetary or non-monetary remuneration or benefits were offered for participation in the study, reducing the potential for participation bias. All data were collected anonymously, further protecting participant confidentiality.

### Statistical analysis

Data collection and entry procedures were conducted using Google Forms. The data was initially entered using Microsoft Excel and then converted into SPSS format for analysis. Using SPSS 25 software, both descriptive and inferential statistical analyses were performed. Descriptive statistics included frequencies, percentages, mean, median, and standard deviation (SD). Inferential statistics involved chi-square tests and logistic regression to identify associations, where research literacy level was considered the outcome variable. All analyses were two-tailed, and a significance level of 0.05 was adopted for all statistical tests, with 95% confidence intervals reported for logistic regression.

To further explore complex associations and enhance prediction, a range of supervised machine learning algorithms, including Logistic Regression, K-Nearest Neighbor, Random Forest, Gradient Boosting Machines, Extreme Gradient Boosting, and Categorical Boosting, were employed using Python in Google Colab. ML was selected to detect potential non-linear relationships and high-order interactions that traditional statistics may miss, providing greater predictive insight. Default hyperparameters were employed due to the study’s exploratory nature; this limitation is acknowledged. Categorical variables were encoded as appropriate. Feature selection was guided by domain expertise, SHAP values, and Cramer’s V, improving model interpretability and identifying key predictors.

A spatial analysis of research literacy was conducted using R statistical software (version 4.3.1; https://www.r-project.org/). Geographic data and shapefiles were obtained from the ‘bangladesh’ package (version 1.0.0; https://cran.r-project.org/package=bangladesh), enabling visualization of research literacy across the country’s eight administrative divisions. GIS-based mapping was used to identify and illustrate regional patterns in research literacy that may not be apparent in overall aggregated analyses. Differences in research literacy by division were assessed using the chi-square test; all spatial analyses were considered exploratory due to lack of statistically significant regional differences. *Post-hoc* stratified analyses were conducted by thesis status (thesis vs. non-thesis group), and the results were visualized on division-level maps generated in R.

### Machine learning models

#### Logistic regression (LR)

A popular statistical technique for binary classification consists of logistic regression, which simulates the likelihood of a binary result depending on one or more predictor factors. LR is appropriate for classification tasks because, in contrast to linear regression, which predicts continuous outcomes, it makes use of the logistic function to guarantee that the predicted probabilities fall between 0 and 1. In addition to being easy to understand and straightforward, this method works well with data that can be divided into linear classes. It may also be used to multiclass situations using strategies such as multinomial logistic regression or one-versus-rest logistic regression^27^.

#### K-nearest neighbor (KNN)

The machine learning technique that is primarily utilized for classification problems is known as the KNN or the distance-based algorithm. To identify which class a new unknown data point belongs to, it seeks to locate all of its nearest neighbors surrounding it. In the feature space, KNN finds the K training instances that are closest to the object that has to be categorized. A majority vote among the object’s K closest neighbors determines the object’s class membership; K is frequently a small positive integer. The object is placed in the class of its closest neighbor when K = 1^29^.

#### Random forest (RF)

Based on classification and decision regression trees, RF is an ensemble machine learning technique. Its foundations are two well-known ensemble learning techniques for classification trees: bagging and boosting^30^. An ensemble technique, which averages or votes among the predictions of several trees, considerably reduces the probability of overfitting, a major limitation in single-decision tree models. Numerous domains have showcased RF’s remarkable precision and versatility, indicating its worth in handling intricate datasets containing a combination of categorical and numerical factors^30^.

#### Gradient boosting machines (GBM)

GBM is an effective ensemble machine learning technology that produces a strong learner by adding weak learners (usually decision trees) one after the other^31^. As a result, predicted accuracy increases. This method repeatedly concentrates on areas where earlier models underperformed by utilizing gradient descent to minimize the loss function. For a variety of predictive applications, GBMs are adaptable and efficient; nevertheless, careful hyperparameter adjustment is needed to avoid overfitting and control computing requirements. They are frequently employed in many different industries due to their capacity to manage complicated, nonlinear data successfully^32^.

#### Extreme gradient boosting (XGBoost)

XGBoost is an ensemble machine learning algorithm that relies on decision trees and employs a gradient-boosting framework^33^. Even though both XGBoost and GBMs use ensemble tree techniques, XGBoost enhances the GBM framework by optimizing systems and improving algorithms. Decision trees are generated sequentially in this approach^32^.

#### Categorical boosting (CatBoost)

A state-of-the-art machine learning technique called CatBoost excels in processing categorical data. CatBoost is a novel technique created by Yandex that reduces typical problems with categorical data without requiring a large amount of pre-processing^34^. This is accomplished by fusing one-hot encoding with an innovative algorithmic method that lowers overfitting and increases prediction accuracy. CatBoost is a widely valued tool for a wide range of applications, including recommendation systems and predictive modeling, because to its well-known efficiency and scalability. The approach is useful in the developing field of machine learning because of its well-documented contribution to improving model performance, especially in datasets with significant categorical characteristics^35^.

### Feature selection

**Fig. 1:**
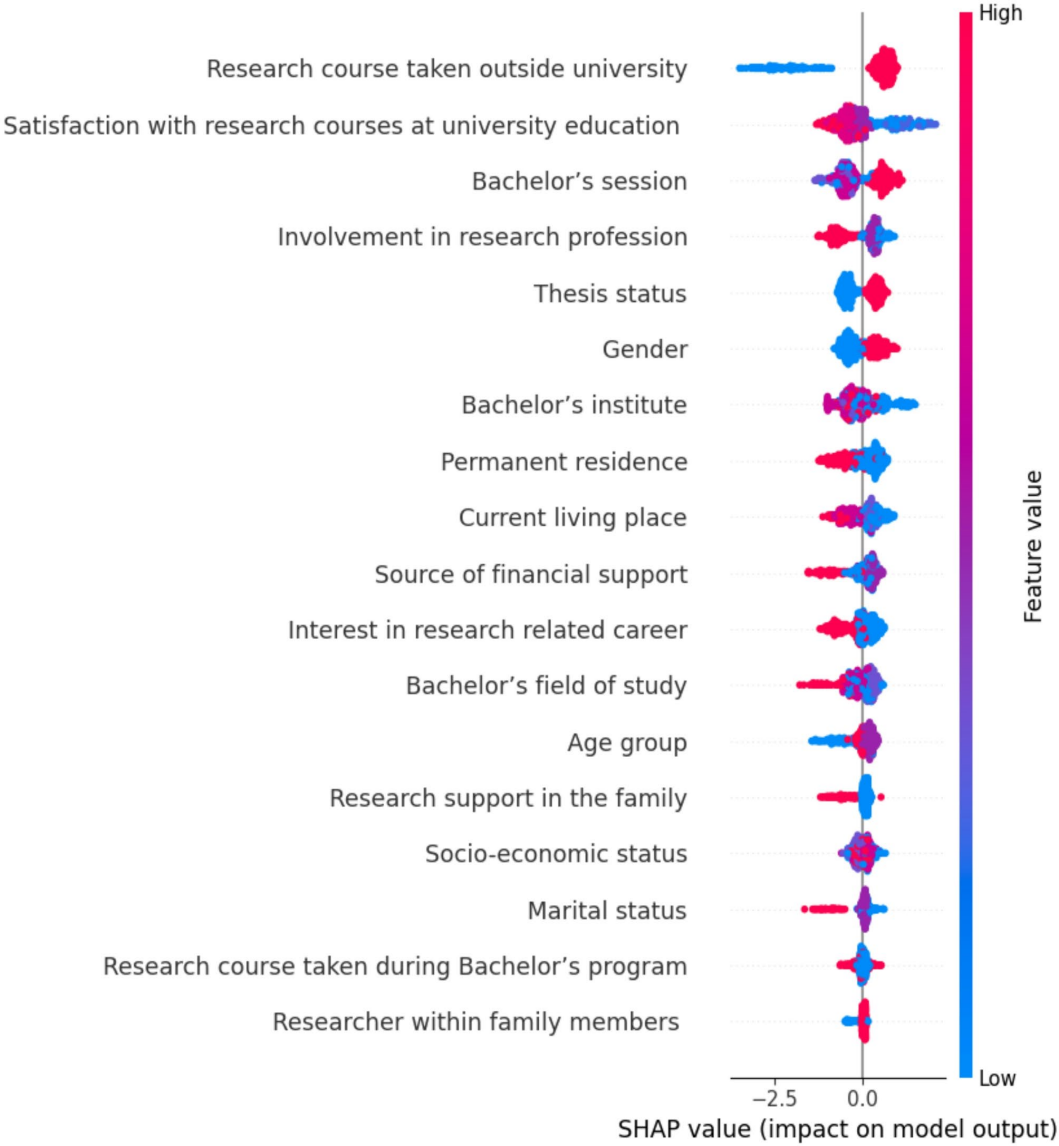
Relative impact of individual features on research literacy predictions, ranked by SHAP values from the XGBoost model. *SHAP (Shapley Additive Explanations)* values represent each feature’s contribution to model output, with higher absolute SHAP values indicating greater influence on predicting research literacy category. XGBoost: Extreme Gradient Boosting. Feature value is color-coded (red: high value, blue: low value).

In the development of machine learning model, an evaluation using XGBoost SHAP values, it is clear that certain features have a more significant impact on the model’s predictions. To ensure robustness and interpretability of the machine learning model, scrutinized the potential predictors using SHAP values to evaluate their impact on model output, and employed Cramer’s V for assessing feature correlations. Research course taken outside university as having the most substantial impact on model outcomes, whereas researcher within family members held minimal influence (Fig. 1). Higher predictive importance was observed in features such as satisfaction with research courses at university education, bachelor’s session, involvement in research profession, thesis status, gender and bachelor’s institute exhibited relatively higher influence compared to other variables. Research course taken outside university high importance likely stems from its role as a strong proxy for a student’s proactive engagement and intrinsic motivation. Unlike standard university curriculum, pursuing external research courses indicates a level of commitment and passion that goes beyond basic requirements. These courses often provide specialized, hands-on skills directly applicable to our outcome variable, making this feature a powerful predictor.

## Results

### Characteristics of the participants

A total of 508 respondents were included in the final analysis, with a mean age of 25.61 years (± 3.124); over half were aged between 24 and 26 years. Males constituted 55.7% of the sample, and 68.9% were single (unmarried or divorced; note, a single response was for divorced). A majority, 61.4%, came from rural families, while 38.6% were from urban areas. Predominantly, 39.2% reported living with their family. Financially, 44.4% received support from their families, and 32% reported low personal income level. In terms of socio-economic status, 29.9% belonged to middle-class families. Nearly four-fifths reported having family support for continuing research, with 16.1% indicating that a family member was engaged in research. Specifically, brothers were the most frequently mentioned (35.9%), followed by sisters (19.2%), spouses (17.9%), and parents (12.8%) (Table 1).

**Table 1:** Descriptions of socio-demographics and its associations with research literacy.

Variables	Total (*n*; %)	Research Literacy		Chi-square Test Statistics		
		Higher Level	Lower Level	χ^2^ test value	df	*p*-value
Gender						
Male	283; 55.7%	149; 52.7%	134; 47.3%	3.754	1	0.053
Female	225; 44.3%	99; 44.0%	126; 56.0%			
**Age group**						
19–23 years	97; 19.1%	36; 37.1%	61; 62.9%	6.594	2	0.037
24–26 years	274; 53.9%	142; 51.8%	132; 48.2%			
27 and above	137; 27.0%	70; 51.1%	67; 48.9%			
**Marital status**						
Married	120; 23.6%	56; 46.7%	64; 53.3%	8.134^a^	2	0.017
Unmarried/Single	350; 68.9%	165; 47.1%	185; 52.9%			
In a relationship	38; 7.5%	27; 71.1%	11; 28.9%			
**Permanent residence**						
Rural	312; 61.4%	143; 45.8%	169; 54.2%	2.885	1	0.089
Urban	196; 38.6%	105; 53.6%	91; 46.4%			
**Current living place**						
With family	199; 39.2%	99; 49.7%	100; 50.3%	3.638	3	0.303
With friends	88; 17.3%	35; 39.8%	53; 60.2%			
In dormitory	177; 34.8%	91; 51.4%	86; 48.6%			
Other places	44; 8.7%	23; 52.3%	21; 47.7%			
**Source of financial support**						
Own	176; 35.1%	89; 50.6%	87; 49.4%	11.598	2	0.003
Family	223; 44.4%	92; 41.3%	131; 58.7%			
Both	103; 20.5%	63; 61.2%	40; 38.8%			
**Socio-economic status**						
Lower Class	98; 20%	44; 44.9%	54; 55.1%	3.931	3	0.269
Middle Class	147; 29.9%	68; 46.3%	79; 53.7%			
High Class	126; 25.7%	71; 56.3%	55; 43.7%			
Upper High Class	120; 24.4%	57; 47.5%	63; 52.5%			
**Personal income level**						
Low Income	95; 32.0%	53; 55.8%	42; 44.2%	5.306	3	0.151
Middle Income	43; 14.5%	27; 62.8%	16; 37.2%			
High Income	90; 30.3%	39; 43.3%	51; 56.7%			
Upper High Income	69; 23.2%	37; 53.6%	32; 46.4%			
**Research support in the family**						
Yes	405; 79.7%	199; 49.1%	206; 50.9%	0.080	1	0.777
No	103; 20.3%	49; 47.6%	54; 52.4%			
**Researcher within family members**						
Yes	82; 16.1%	48; 58.5%	34; 41.5%	3.696	1	0.055
No	426; 83.9%	200; 46.9%	226; 53.1%			
**Researcher within family members: Relationship**						
Spouse	14; 17.9%	6; 42.9%	8; 57.1%	5.908	4	0.206
Parent	10; 12.8%	4; 40.0%	6; 60.0%			
Brother	28; 35.9%	19; 67.9%	9; 32.1%			
Sister	15; 19.2%	11; 73.3%	4; 26.7%			
Others	11; 14.1%	5; 45.5%	6; 54.5%			

Notes: df = degrees of freedom; p-value = probability value; χ² test value = Chi-square statistic. “Higher Level” and “Lower Level” refer to research literacy categorized using the median split. Socio-economic status and personal income levels are based on self-reported monthly income. “Other” in Relationship includes uncle, cousin, brother-in-law, sister-in-law, father-in-law, etc. Percentages may not sum to 100% due to rounding.

### Descriptions of academic information

Regarding academic backgrounds, 37.1% of participants attended general universities for their bachelor’s degrees, with 35.1% focusing on social sciences and arts. Besides, 54.4% reported pursuing or having completed a bachelor’s and master’s degree from the same university. Among those with master’s degrees, 44.7% studied at general universities, and 32.8% focused on social sciences and arts. In terms of academic performance, 41.6% reported a bachelor’s CGPA between 3.50 and 3.75, while 35.6% had a master’s CGPA of 3.75 or above. Furthermore, 58.1% of respondents experienced session jams during their academic programs (Table 2).

**Table 2:** Descriptions of academic and research training-related variables and their associations with research literacy.

Variables	Total (*n*; %)	Research Literacy		Chi-square Test Statistics		
		Higher Level	Lower Level	χ^2^ test value	df	*p*-value
Education related iInformation						
**Bachelor’s institute**						
General	187; 37.1%	96; 51.3%	91; 48.7%	3.692	3	0.297
Private	62; 12.3%	29; 46.8%	33; 53.2%			
Specialized	153; 30.4%	80; 52.3%	73; 47.7%			
Health Sciences	102; 20.2%	42; 41.2%	60; 58.8%			
**Bachelor’s field of study**						
Life Sciences	156; 30.8%	80; 51.3%	76; 48.7%	2.804	3	0.423
Health Sciences	134; 26.4%	57; 42.5%	77; 57.5%			
Social Sciences and Arts	178; 35.1%	90; 50.6%	88; 49.4%			
Business and others	39; 7.7%	20; 51.3%	19; 48.7%			
**Bachelor’s session**						
Before 2014-15	70; 15.5%	39; 55.7%	31; 44.3%	20.322	2	< 0.001
2015-16 to 2017-18	186; 41.1%	109; 58.6%	77; 41.4%			
2018-19 and above	197; 43.5%	72; 36.5%	125; 63.5%			
**Bachelor’s and Master’s from the same institute**						
Yes	223; 54.4%	131; 58.7%	92; 41.3%	5.086	1	0.024
No	187; 45.6%	89; 47.6%	98; 52.4%			
**Master’s institute**						
General	119; 44.7%	59; 49.6%	60; 50.4%	2.148	3	0.542
Private	34; 12.8%	18; 52.9%	18; 47.1%			
Specialized	94; 35.3%	54; 57.4%	40; 42.6%			
Health Sciences	19; 7.1%	08; 42.1%	11; 57.9%			
**Bachelor’s and Master’s from different institute**						
General	15; 23.4%	7; 46.7%	8; 53.3%	2.815	5	0.729
Private	26; 40.6%	13; 50.0%	13; 50.0%			
Medical	5; 7.8%	3; 60.0%	2; 40.0%			
Foreign Based University	5; 7.8%	4; 80.0%	1; 20.0%			
Agricultural	7; 10.9%	5; 71.4%	2; 28.6%			
Others	6; 9.4%	3; 50.0%	3; 50.0%			
**Master’s field of study**						
Sciences	87; 36.1%	55; 63.2%	32; 36.8%	2.262	3	0.520
Health Sciences	52; 21.6%	27; 51.9%	25; 48.1%			
Social Sciences	79; 32.8%	50; 63.3%	29; 36.7%			
Business and others	23; 9.5%	13; 56.5%	10; 43.5%			
**Master’s session**						
Before 2021	64; 27.6%	36; 56.3%	28; 43.8%	0.494	1	0.482
After 2021	168; 72.4%	103; 61.3%	65; 38.7%			
**Session jam during university education**						
Yes	272; 58.1%	149; 54.8%	123; 45.2%	6.477	1	0.011
No	196; 41.9%	84; 42.9%	112; 57.1%			
**Bachelor’s results (GPA/CGPA)**						
Below 3.25	75; 21.1%	39; 52.0%	36; 48.0%	3.151	3	0.369
3.25–3.49	87; 24.4%	50; 57.5%	37; 42.5%			
3.50–3.74	148; 41.6%	77; 52.0%	71; 48.0%			
3.75- Above	46; 12.9%	19; 41.3%	27; 58.7%			
**Master’s results (GPA/CGPA)**						
Below 3.50	35; 33.7%	20; 57.1%	15; 42.9%	2.277	2	0.320
3.50–3.74	32; 30.8%	19; 59.4%	13; 40.6%			
3.75 – Above	37; 35.6%	27; 73.0%	10; 27.0%			
**Research Courses/Training Related Information**						
**Research course taken during Bachelor’s program**						
Yes	372; 73.2%	191; 51.3%	181; 48.7%	3.546	1	0.060
No	136; 26.8%	57; 41.9%	79; 58.1%			
**Research course taken during Master’s program**						
Yes	207; 73.9%	128; 61.8%	79; 38.2%	3.487	1	0.062
No	73; 26.1%	36; 49.3%	37; 50.7%			
**Satisfaction with research courses at university education**						
Not at all	42; 9.9%	11; 26.2%	31; 73.8%	24.457	3	< 0.001
Somewhat	161; 38.1%	73; 45.3%	88; 54.7%			
Moderately	165; 39.0%	96; 58.2%	69; 41.8%			
Highly	55; 13.0%	39; 70.9%	16; 29.1%			
**Research course taken outside university**						
Yes	99; 19.5%	81; 81.8%	18; 18.2%	53.590	1	< 0.001
No	409; 80.5%	167; 40.8%	242; 59.2%			
**Number of other research courses taken outside university**						
One course	41; 53.9%	35; 85.4%	6; 14.6%	0.383	1	0.536
More than one course	35; 46.1%	28; 80.0%	7; 20.0%			
**Satisfaction with other research courses outside university**						
Not at all	7; 7.4%	4; 57.1%	3; 42.9%	11.015	3	0.012
Somewhat	18; 18.9%	11; 61.1%	7; 38.9%			
Moderately	37; 38.9%	31; 83.8%	6; 16.2%			
Highly	33; 34.7%	31; 93.9%	2; 6.1%			

Notes: df = degrees of freedom; p-value = probability value; χ² test value = Chi-square statistic. “Higher Level” and “Lower Level” refer to research literacy categorized by the median split. Bachelor’s institute: General = General and National universities; Specialized = Technology, Engineering, and Agriculture; Health Sciences = Medical, Nursing, Health Sciences, and Public Health. “Session jam” refers to delayed academic sessions during university education. GPA/CGPA = Grade Point Average/Cumulative Grade Point Average (self-reported). Percentages may not sum to 100% due to rounding.

### Descriptions of research courses related to information

Regarding research-related courses, 73.2% of participants reported taking at least one course during their bachelor’s program, and 73.9% during their master’s program. Besides, 19.5% had taken research courses or training programs outside of university, with 53.9% taking just one course. Participants evaluated their university research courses as not at all helpful (9.9%), somewhat helpful (38.1%), moderately helpful (39%), and highly helpful (13%). For courses taken outside the university, the evaluations were 7.4%, 18.9%, 38.9%, and 34.7%, respectively (Table 2).

### Descriptions of research experience and career related to information

Regarding research experience and career information, 52.8% of participants reported completing or nearing completion of their thesis. Besides, 25.2% were currently working on a research project other than their thesis. Among those, 15.8% had published at least one paper, 12.5% had papers under review, and 71.7% were continuing their work. In terms of publication, 57.1% of respondents had more than one journal publication. Furthermore, 55.3% were not involved in a research-related profession, and 60.4% expressed interest in a research-related career. Specifically, 71.0% were interested in research due to plans for higher study, followed by working in the research and development sector (48.3%), building a career as a researcher (40.1%), and pursuing an academic position (37.8%) (Table 3).

**Fig. 2:**
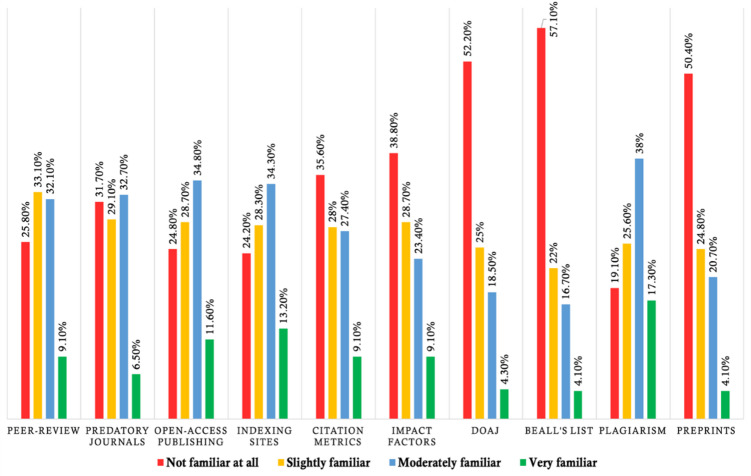
Distribution of research literacy item familiarity among participants. *DOAJ*: Directory of Open Access Journals. “Not familiar at all” = 0, “Slightly familiar” = 1, “Moderately familiar” = 2, “Very familiar” = 3. Percentages reflect self-reported familiarity with each concept. Higher percentages in “Not familiar at all” and “Slightly familiar” indicate knowledge gaps. Items correspond to the ten research literacy domains assessed in the study.

### Research literacy levels

The results of the study revealed significant gaps in research literacy among participants. Specifically, 51.2% of participants were classified as having lower research literacy. Regarding specific areas of knowledge, 25.8% of respondents reported being not familiar with the peer review process, while 31.7% were not familiar with predatory journals. Knowledge of open access publishing was lacking in 24.8% of participants, and 24.2% were not familiar with indexing sites. Citation metrics were unfamiliar to 35.6% of respondents, and 38.8% did not recognize impact factors or site scores. The Directory of Open Access Journals was not familiar to a notable 52.2% of participants, and a majority of 57.1% were not familiar with Beall’s List. Besides, 19.1% of respondents lacked awareness of plagiarism, and 50.4% were not familiar with the concept of preprints (Fig. 2).

### Associations between the study variables and research literacy

Significant differences in research literacy levels were observed across various demographic and academic variables. In terms of gender, no difference was observed in research literacy levels, with 56.0% of females and 47.3% of males reporting lower literacy (χ^2^ = 3.754, *p* = 0.053). Younger students (ages 19–23) exhibited lower research literacy compared to older groups (χ^2^ = 6.594, *p* = 0.037). Marital status also played a role, with married or single individuals more likely to report lower literacy than those in a relationship (χ^2^ = 8.134, *p* = 0.017). Financial support was another factor, as those receiving family support had a higher proportion of lower research literacy compared to those with personal or combined support (χ^2^ = 11.598, *p* = 0.003). (Table 1).

About 52.4% of those who pursued both bachelor’s and master’s degrees at different institutions had lower research literacy compared to those from the same institution (; χ^2^ = 5.086, *p* = 0.024). Participants who did not experience academic session interruptions or delay were more likely to have lower research literacy than those who did ( χ^2^ = 6.477, *p* = 0.011). External research training positively impacted research literacy. Students who took no training outside the university had lower literacy (59.2%), whereas only 18.2% of those with external training reported lower literacy. Evaluations of research courses as unsatisfactory correlated with lower literacy levels for both university courses (χ^2^ = 24.457, *p* < 0.001) and external courses (χ^2^ = 11.015, *p* = 0.012) **(**Table 2**)**.

**Table 3:** Descriptions of research experience and career-related variables and their associations with research literacy.

Variables	Total (*n*; %)	Research Literacy		Chi-square Test Statistics		
		Higher Level	Lower Level	χ^2^ test value	df	*p*-value
Research Experience and Publication Related Information						
**Thesis status**						
Thesis group	268; 52.8%	159; 59.3%	109; 40.7%	25.076	1	< 0.001
Non-thesis group	240; 47.2%	89; 37.1%	151; 62.9%			
**Research experience except thesis**						
Yes	128; 25.2%	102; 79.7%	26; 20.3%	64.888	1	< 0.001
No	379; 74.8%	146; 38.5%	233; 61.5%			
**Research experience except thesis: status**						
Continuing work, will submit in journal	86; 71.7%	66; 76.7%	20; 23.3%	3.149	2	0.207
Submitted to journal, under-review	15; 12.5%	12; 80.0%	3; 20.0%			
Published at least a paper	19; 15.8%	18; 94.7%	1; 5.3%			
**Number of publications for someone engaged with research**						
One publication	18; 42.9%	13; 72.2%	5; 27.8%	0.754	1	0.385
More than one publication	24; 57.1%	20; 83.3%	4; 16.7%			
**Research Career Related Variables**						
**Involvement in research related profession**						
No research related job	230; 55.3%	80; 34.8%	150; 65.2%	50.711	1	< 0.001
Research related job	186; 44.7%	130; 69.9%	56; 30.1%			
**Interest in research related career**						
Yes	307; 60.4%	162; 52.8%	145; 47.2%	4.845	1	0.028
No	201; 39.6%	86; 42.8%	115; 57.2%			
**Reasons for choosing research as career** (multiple responses)						
*Interest in academic job or university faculty*	124; 37.8%	67; 54.0%	57; 46.0%	0.037	1	0.848
*Interest in higher study*	233; 71.0%	128; 54.9%	105; 45.1%	0.809	1	0.368
*Working at development sector*,* NGOs*	119; 36.3%	71; 59.7%	48; 40.3%	2.988	1	0.084
*Building career as a researcher*	131; 40.1%	78; 59.5%	53; 40.5%	3.189	1	0.074
*Working at research and development sector*	157; 48.3%	86; 54.8%	71; 45.2%	0.292	1	0.589
*Gaining professional recognition*	83; 25.6%	49; 59.0%	34; 41.0%	1.276	1	0.259
*Requirement of institution or program*	19; 5.8%	11; 57.9%	8; 42.1%	0.166	1	0.684
*Gaining personal recognition*	82; 25.0%	53; 64.6%	29; 35.4%	5.590	1	0.018
*Interest in interdisciplinary collaboration and networking*	56; 17.1%	33; 58.9%	23; 41.1%	0.843	1	0.358
*Developing research skills*	174; 53.0%	99; 56.9%	75; 43.1%	1.869	1	0.172

df = degrees of freedom; p-value = probability value; χ² test value = Chi-square statistic. “Higher Level” and “Lower Level” refer to research literacy categorized by the median split. “Thesis group” includes those with a completed or ongoing thesis; “Non-thesis group” includes those unwilling, undecided, or without the opportunity to do a thesis. “Research experience except thesis” refers to participation in research projects other than mandatory thesis work. “Research related profession” includes employment in academic, research, or related sectors; “No research related job” includes those not engaged in research employment. “Reasons for choosing research as career” were multiple response options; percentages are calculated out of those expressing interest in a research career and may sum to more than 100%. Percentages may not total 100% due to rounding or multiple response options.

Thesis completion status also mattered; non-thesis group members had significantly lower research literacy than thesis group members ( χ^2^ = 25.076, *p* < 0.001). Those not involved in any research activity without a thesis had higher rates of lower research literacy than those involved in research work without a thesis (; χ^2^ = 64.888, *p* < 0.001). Similarly, respondents not engaged in any research-related jobs reported lower literacy levels compared to those who were ( χ^2^ = 50.711, *p* < 0.001). However, motivation to engage in research was significantly associated with literacy levels. Lower literacy was more common among those not interested in research careers ( *p* = 0.028). Specific motivations such as personal recognition ( χ^2^ = 5.590, *p* = 0.018), were linked to higher research literacy (Table 3).

**Table 4:** Binary logistic regression analysis of the variables with research literacy.

Variables	Unadjusted Model					Adjusted Model				
	Odds Ratio (OR)	95% Confidence Interval (CI)			*p*-value	Adjusted Odds Ratio (AOR)		95% Confidence Interval (CI)		*p*-value
Gender										
Male	0.707	0.497–1.005			0.053	0.580		0.314–1.070		0.081
Female	Reference					Reference				
**Age group**										
19–23 years	1.770	1.041–3.010			0.039	0.365		0.088–1.516		0.287
24–26 years	0.971	0.644–1.464				0.828		0.302–2.275		
27 and above	Reference					Reference				
**Marital status**										
Married	2.805	1.276–6.165			0.022	2.318		0.600–8.952		0.376
Unmarried/Single	2.752	1.324–5.721				2.317		0.708–7.584		
In a relationship	Reference					Reference				
**Permanent residence**										
Rural	1.364	0.953–1.951			0.090	1.792		0.964–3.332		0.065
Urban	Reference					Reference				
**Current living place**										
Family	1.106	0.575–2.127			0.307	1.296		0.416–4.035		0.393
Friends	1.659	0.800–3.440				2.420		0.643–9.106		
Dormitory	1.1035	0.534–2.004				1.128		0.321–3.970		
Others	Reference					Reference				
**Source of financial support**										
Own	1.540	0.939–2.524			0.003	2.115		0.881–5.077		0.084
Family	2.243	1.391–3.616				2.338		1.066–5.130		
Both	Reference					Reference				
**Socio-economic status**										
Lower Class	1.110	0.650–1.897			0.272	0.791		0.310–2.017		0.781
Middle Class	1.051	0.648–1.704				0.868		0.379–1.990		
High Class	0.701	0.424–1.158				0.656		0.286–1.504		
Upper High Class	Reference					Reference				
**Research support in the family**										
Yes	0.939	0.609–1.448			0.777	1.546		0.681–3.507		0.298
No	Reference					Reference				
**Researcher within family members**										
Yes	0.627	0.388–1.012			0.056	0.939		0.426–2.072		0.877
No	Reference					Reference				
**Bachelor’s institute**										
General	0.644	0.407–1.080			0.299	1.177		0.271–5.117		0.990
Private	0.797	0.422–1.504				1.004		0.200–5.029		
Specialized	0.639	0.385–1.060				1.225		0.228–6.591		
Health Sciences	Reference					Reference				
**Bachelor’s field of study**										
Life Sciences	1.000	0.496–2.018			0.425	1.564		0.508–4.814		0.176
Health Sciences	1.422	0.695–2.907				4.404		0.884–21.940		
Social Sciences and Arts	1.029	0.515–2.059				2.662		0.835–8.483		
Business and others	Reference					Reference				
**Bachelor’s session**										
Before 2014-15	0.458	0.263–0.796			< 0.001	0.163		0.041–0.645		< 0.001
2015-16 to 2017-18	0.407	0.270–0.614				0.176		0.079–0.394		
2018-19 to above	Reference					Reference				
**Research course taken during bachelor’s program**										
Yes	0.684	0.460–1.017			0.060	0.625		0.289–1.352		0.233
No	Reference					Reference				
**Satisfaction with research courses at university education**										
Not at all	6.869	2.790–16.912			< 0.001	11.979		3.000–47.825		< 0.001
Somewhat	2.938	1.520–5.682				2.460		0.933–6.489		
Moderately	1.752	0.906–3.386				0.962		0.362–2.555		
Highly	Reference					Reference				
**Research course taken outside university**										
Yes	0.153	0.089–0.265			< 0.001	0.166		0.066–0.417		< 0.001
No	Reference					Reference				
**Thesis status**										
Thesis group	0.404			0.283–0.578	< 0.001	0.671	0.357–1.260			0.214
Non-thesis group	Reference					Reference				
**Interest in research related career**										
Yes	0.669	0.468–0.958			0.028	0.733		0.379–1.416		0.355
No	Reference					Reference				
**Involvement in research profession**										
No	4.353		2.876–6.588		< 0.001	3.944			2.016–7.717	< 0.001
Yes	Reference					Reference				

Notes: OR = Odds Ratio; AOR = Adjusted Odds Ratio; CI = Confidence Interval. “Unadjusted Model” presents crude (univariate) associations; “Adjusted Model” presents multivariable associations, adjusted for all variables listed. For categorical variables, the “Reference” group is the comparison category.

### Factors associated with research literacy

Table 4 presents the results of logistic regression analysis examining factors associated with lower research literacy levels. In the adjusted model, several significant associations were identified. Students admitted after 2018-19 in their bachelor’s program were found to be at a higher risk of lower literacy compared to those who graduated earlier. Furthermore, students who rated their research courses taken for academic requirements as unsatisfactory were 11.979 times more likely to have lower literacy levels, while those who expressed somewhat satisfactory were at 2.46 times higher risk of lower literacy compared to those who are highly satisfied. Besides, students who did not engage in research courses or training outside their university had a significantly higher risk of lower literacy (aOR = 0.166, 95% CI = 0.066–0.417, *p* < 0.001). Finally, individuals not involved in research-related professions were nearly four times more likely to have lower literacy levels compared to their counterparts (Table 4).

**Fig. 3:**
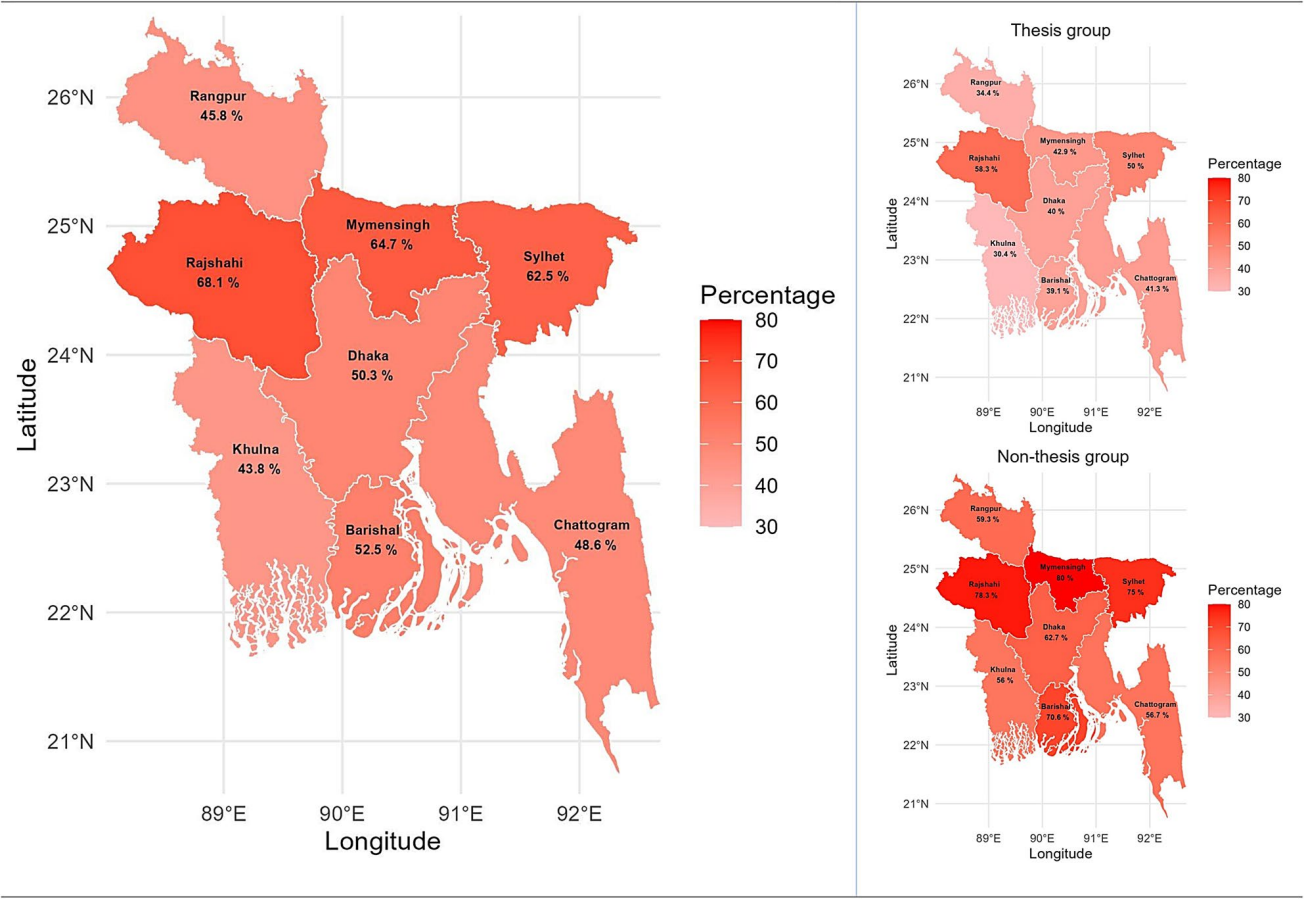
Geographic distribution of low research literacy among participants by division and thesis status in Bangladesh. Panel A (left) displays the percentage of students/graduates with low research literacy in each of the eight divisions. Panels B and C (right) show the percentages stratified by thesis group and non-thesis group, respectively. Percentages are color-coded (darker shades indicate higher proportions of low research literacy). Maps were created using the R ‘bangladesh’ package, based on self-reported division of residence. Thesis group: completed or ongoing thesis. Non-thesis group: undecided, unwilling, or no opportunity for thesis.

Map created using R statistical software (version 4.3.1, https://www.r-project.org/) and the ‘bangladesh’ package (version 1.0.0, https://cran.r-project.org/package=bangladesh).

### Regional distributions of research literacy

The GIS-based visualization in Fig. 3 illustrates the distribution of research literacy levels across different regions. While the distribution of lower research literacy was not significantly associated with division overall (χ^2^ = 9.234, *p* = 0.236), certain divisions, such as Rajshahi, Mymensingh, and Sylhet, exhibited higher rates of lower literacy. Similarly, when considering student status (thesis vs. non-thesis), no significant difference was observed (thesis group: χ^2^ = 4.837, *p* = 0.680; non-thesis group: χ^2^ = 6.024, *p* = 0.537). However, it is noteworthy that thesis students from the Rajshahi division showed a notably higher prevalence of lower literacy, while for the non-thesis group, it was observed in Rajshahi, Mymensingh, Barishal, and Sylhet (Fig. 3).

### Evaluation of machine learning model performances

Table 5 displays the performance metrics of the algorithms. With the greatest accuracy scores of 73.04% and 71.57% for research literacy, the Random Forest and CatBoost algorithms, respectively fared notably better than the other algorithms. Similarly, RF also outperformed in precision scores is 73.29%, where CatBoost achieved 71.69%. In terms of F1-score, CatBoost showed good performance scores of 71.60%, but RF achieved the highest score 73.08%. Furthermore, every algorithm preserved good logarithmic loss values below 2%. The CatBoost and RF model’s overall superior performance across metrics highlights this predictive analysis’s effectiveness (Table 5).

**Table 5:** Evaluation of machine learning model performances.

Model	ACC	Precision	F1 score	Logg loss
**KNN**	58.82	58.58	58.56	1.33
**RF**	73.04	73.29	73.08	0.57
**XGBoost**	70.59	70.80	70.63	0.73
**GBM**	68.63	68.56	68.57	0.61
**CatBoost**	71.57	71.69	71.60	0.56

Notes: ACC = Accuracy (%); F1 score = Harmonic mean of precision and recall (%); Logg loss = Logarithmic loss (lower values indicate better model fit). KNN = K-Nearest Neighbor; RF = Random Forest; XGBoost = Extreme Gradient Boosting; GBM = Gradient Boosting Machine; CatBoost = Categorical Boosting. Model performances were assessed using hold-out test data (or specify cross-validation if used). Higher ACC, Precision, and F1 scores, and lower Logg loss indicate better predictive performance.

**Fig. 4:**
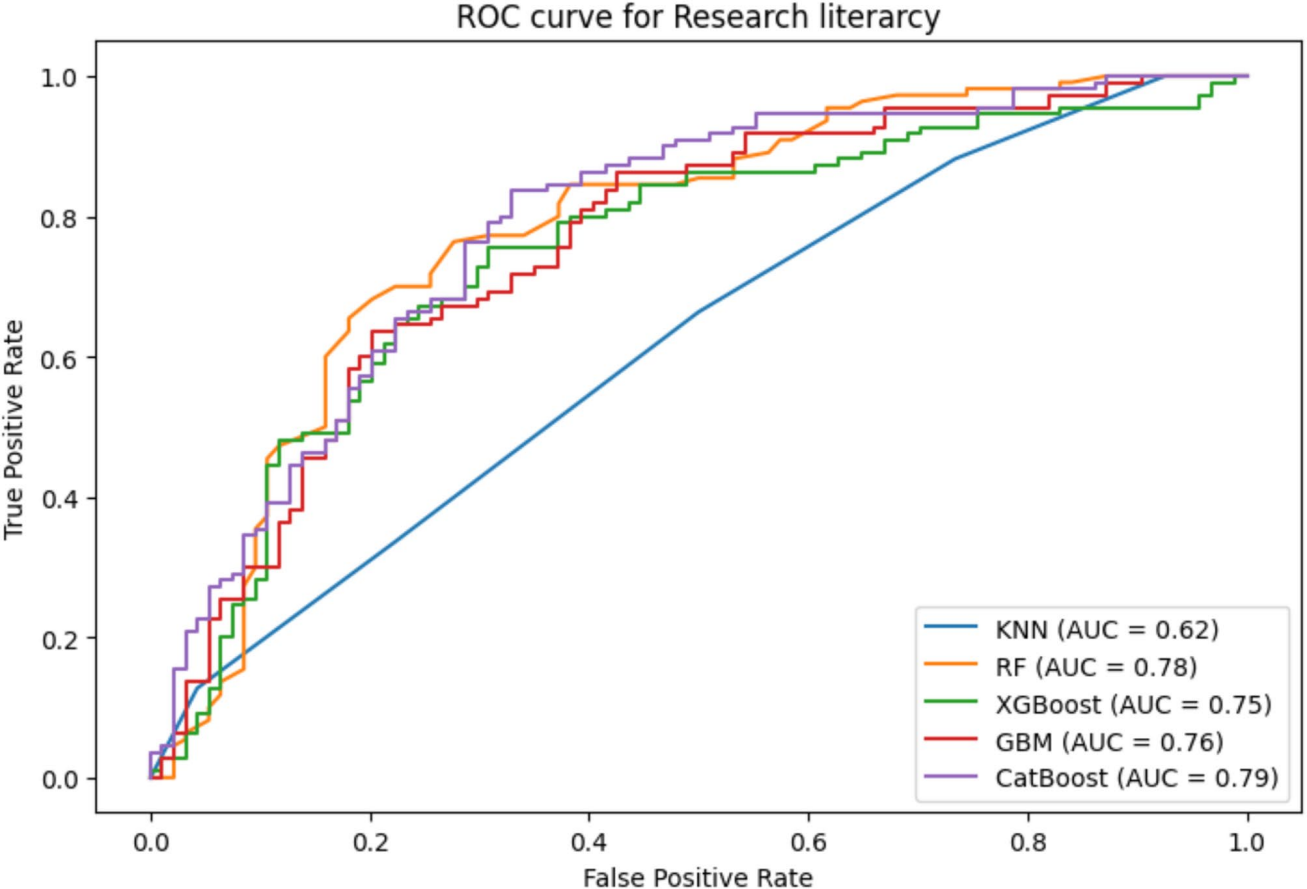
Receiver Operating Characteristic (ROC) curves for machine learning models predicting research literacy status. Curves represent the classification performance of K-Nearest Neighbor (KNN), Random Forest (RF), Extreme Gradient Boosting (XGBoost), Gradient Boosting Machine (GBM), and Categorical Boosting (CatBoost) algorithms. The Area Under the Curve (AUC) values for each model are provided in the legend. Higher AUC indicates better model discrimination between high and low research literacy.

The Area Under the Receiver Operating Characteristic Curve (ROC-AUC) is a commonly used statistic in machine learning to evaluate the effectiveness of binary classification models, measuring how well they can distinguish between positive and negative classifications. The models for research literacy, CatBoost and RF achieved the greatest AUC scores of 0.79 and 0.78, respectively indicating their remarkable accuracy in differentiating between positive and negative classifications (Fig. 4). This highlights the accuracy of the models in using estimated probability to rank cases from the two classes. In this analysis, the AUC of the KNN model is marginally lower at 0.62. According to the AUC value, the CatBoost model stands out for having the strongest discriminatory power among the models, all of which show a respectable level of skill in differentiating between positive and negative classes.

## Discussion

This study is the first to comprehensively investigate research literacy levels among university students and graduates, whereby several factors associated with poor research literacy are identified. We also incorporated GIS and Machine Learning applications to provide more holistic findings. The results indicate that more than half of the participants lack sufficient knowledge in recognizing various aspects related to research and scholarly publications. Factors such as satisfaction with research courses at university education, research course taken outside university, and professional engagement in research were significant determinants of research literacy. Feature selection analysis conducted through machine learning revealed research courses taken outside the university had the most significant influence on predicting literacy, but that presence of researchers within the family had the least impact. In addition, regional variations were not significant in terms of research literacy distribution, as found in GIS analyses. While some patterns in research literacy are consistent with prior studies, our multidimensional approach and use of advanced analytics revealed several novel insights. Most notably, the central role of external research training, the limited influence of regional factors, and the identification of persistent gaps in awareness of modern publishing tools and platforms all provide fresh directions for educational policy and practice, both in Bangladesh and beyond.

The proliferation of predatory journals poses a significant threat to academic integrity, as these outlets often bypass rigorous peer review, lack transparent editorial processes, and enable the spread of low-quality or even unethical research^18,36^. In highly competitive “*publish or perish*” environments, early-career researchers and students may feel institutional pressure to publish quickly, which increases their vulnerability to such journals—particularly when research literacy or mentorship is limited^7,15,18^. Engagement with predatory publishers can result in wasted time and resources, undermined reputations, and stalled career advancement, as these publications are often not recognized by reputable databases and may disqualify individuals from grants, promotions, or academic positions^11,37^. Furthermore, the spread of unreliable or fraudulent findings through predatory journals erodes public trust in science and can distort the evidence base for decision-making in health and policy^12^. Our findings, consistent with those of prior studies, highlight the importance of equipping students and graduates with the knowledge and critical skills to recognize, avoid, and report predatory publishing practices^11,15,18,38^. This is particularly urgent in settings where rapid publication is prioritized over rigorous scholarship, further highlighting the need for robust research literacy education, institutional policies, and mentorship structures to protect both individual researchers and the broader scientific community.

This study reveals that a majority of the participants demonstrate poor research literacy levels. More specifically, 25.8% of the participants had not heard of the peer-review process, 31.7% could not recognize predatory journals, 24.8% were unaware of open-access publishing, and 24.2% were not familiar with indexing sites. Furthermore, 35.6% had no knowledge of citation metrics, 38.8% were unfamiliar with impact factors, and 52.2% and 57.1% had never heard of the Directory of Open Access Journals (DOAJ) and Beall’s List, respectively. Besides, 19.1% lacked understanding of plagiarism, and 50.4% were unfamiliar with preprints. Comparative findings from previous studies show similar trends. For instance, a lack of literacy and inability to identify predatory journals were observed among dermatologists in Austria^14^ and among academic nurse researchers^9^. Both groups published in predatory journals due to poor understanding of predatory journals and a lack of research literacy training. In these studies, participants could not identify predatory journals, with three-quarters of nurses and 70.6% of dermatologists unable to do so. Furthermore, 93.3% of dermatologists were unaware of Beall’s List, and 43.7% were unfamiliar with open-access publishing^14^. A study among German orthopedic and trauma surgeons found that 79% of participants had not heard of DOAJ^15^. In a comparative study involving medical students from Saudi Arabia and New Zealand, 30.8% and 42.2%, respectively, were familiar with open-access publishing, and even fewer were aware of predatory journals (9.1% and 7.8%, respectively) or of the Beall’s List (2.5% and 0.0%, respectively)^13^. Al Ryalat et al.^10^ found that 93%, 97.5%, and 10.8% of biomedical researchers were unaware of predatory journals, Beall’s List, and indexing services, respectively. However, a one-minute infographic presentation on these issues significantly improved the understanding by declining these rates to 2.5%, 5.1%, and 21.5%, respectively^10^. While direct cross-national comparisons are limited by differences in measurement and context, our results align with global concerns regarding insufficient research literacy among students and early-career researchers. Several international studies have reported persistent gaps in knowledge of predatory journals, peer review, and research ethics across diverse settings, suggesting that limited research literacy is a widespread challenge, not unique to Bangladesh. The lack of comprehensive worldwide data on research literacy highlights the need for further cross-cultural and multinational investigations to benchmark progress and inform targeted interventions globally.

This study finds that female participants are more likely to have poor knowledge of research (56% vs. 47.3%). This finding aligns with an interventional study among healthcare workers in Jordan, where females were found to have significantly lower familiarity with predatory journals pre-intervention, although post-intervention, the difference became non-significant^26^. Similarly, a study among dermatologists in Austria^14^ revealed that women were more likely oblivious to predatory journals than men. This gender-based disparity in research literacy can be attributed to the different research opportunities available to each gender. In many socio-cultural backgrounds, males are likely to have access to opportunities for exploring and engaging in research-related careers. This systemic bias likely enhances their research literacy compared to their female counterparts.

This study also found a higher level of research literacy among the older age group. Students aged between 19 and 23 had higher odds of poor research literacy compared to those aged 27 or above in the unadjusted model. A similar result was observed in a study among graduate medical education trainees in the United Arab Emirates, where junior trainees were more vulnerable to predatory publishing, indicating lower research knowledge^5^. Younger students in Bangladesh are more likely to face challenges in understanding and conducting research due to factors such as less academic experience, limited access to research resources, inadequate training in research methodologies, or a lack of mentorship at earlier educational stages. Older students usually have more experience with thesis work, and this study showed that the non-thesis group exhibited higher likelihood of lower literacy levels than the thesis group. The progressive increase in knowledge with age is likely associated with increased involvement in research as students’ progress through their educational stages. Interestingly, the adjusted regression model suggested that younger generations might be more enthusiastic and updated on research nowadays. This could be attributed to the rapid technological advancements and the increased exposure to research problems. As a result, their knowledge about research literacy has also been enriched. However, this indicates that research literacy is a complex phenomenon influenced by a combination of factors related to individual demographics, academic background, research exposure, and institutional support.

The study results indicate that 16.1% of all participants had family members who were researchers, and these participants demonstrated higher research literacy. However, the specific relationship of the researcher within the family (e.g., spouses, parents, brothers, sisters, and other relatives) did not significantly differ in terms of research literacy levels. This suggests that having a family member working in research influences an individual’s interest in research, regardless of the specific relationship. This finding aligns with Funk and Hefferon’s statement based on a 2014’s survey^39^, which found that 12% of working PhD scientists in the American Association for the Advancement of Science were primarily motivated by their parents and other family members to pursue careers as scientists. Participants with researchers in their families likely have higher research literacy due to regular exposure to academic and intellectual conversations within the family, as well as better access to educational resources such as books, journals, and other materials, fostering a rich learning environment. Having a researcher in the family can serve as a role model, inspiring other members to pursue knowledge and academic excellence. Family researchers provide valuable guidance and support for intellectual development, helping family members navigate educational challenges more effectively and enhancing their critical thinking and comprehension skills. The support from family members, whether mental, financial, or critical, significantly contributes to higher research literacy among upcoming researchers from these families. This support network creates an environment conducive to academic success, explaining why participants with researchers in their families demonstrate higher research literacy levels. However, it is worth noting that family support in this study was not directly associated with higher research knowledge, which warrants further investigation.

Participants who had taken research courses during their bachelor’s program exhibited significantly 1.6 times higher odds of research literacy than those who did not. This suggests that formal education in research methods during undergraduate studies significantly enhances students’ research literacy. Therefore, integrating research courses into the bachelor’s curriculum is crucial for equipping students with the necessary skills and knowledge to conduct and understand research effectively. Similarly, those who pursued research courses outside of university showed a remarkable 6 times higher literacy level. Enrolling in such courses provides students with a structured curriculum, practical experience, access to resources, mentorship, and collaborative learning, all of which collectively enhance their research literacy. This is further evidenced by the finding that participants who were not satisfied with the research courses were more likely to report poor research knowledge. These findings highlight the critical role of both formal and external research education in developing robust research literacy among students.

Participants enrolled in the thesis group exhibited significantly 2.47 times higher odds of research literacy than those in the non-thesis group in the unadjusted model. Thesis group subjects are more likely to be associated with research due to their formal education requirements. Besides, individuals not involved in research professions have higher odds of poor research literacy compared to those who are involved, highlighting the impact of personal motivation and professional engagement on research knowledge. Involvement in research professions offers practical experience and regular exposure to a research-focused environment, including access to research tools, resources, and mentorship. This engagement, along with ongoing learning and the application of research knowledge in professional settings, reinforces and improves comprehension of research literacy. Moreover, higher research literacy was more evident among participants who had published at least one paper compared to those who were still working on their research. This finding aligns with Richtig et al.^16^, who reported that participants with a higher number of publications and more high-impact publications were more likely to be aware of predatory journals. Similarly, participants who were aware of predatory journals were often listed as corresponding authors and were well-published as first or last authors^15^. Hashish et al.^9^ also reported that faculty members with previous publishing experience were more knowledgeable in distinguishing between predatory and legitimate journals. Thus, it is evident that participants with research exposure and publishing experience are more likely to possess higher research literacy as practical research engagement enhances research knowledge and awareness.

Regional mapping revealed that spatial variation in terms of lower research literacy did not significantly differ by division within Bangladesh. However, individuals from the divisions of Rajshahi, Mymensingh, and Sylhet were found to be more prone to poor literacy levels. It was initially hypothesized that there might be differences in research literacy levels based on thesis status, with those in the thesis group expected to report higher research literacy than their counterparts. However, post-hoc analyses did not show significant variation in spatial distribution of poor literacy levels based on thesis status. Despite the thesis status, divisions such as Rajshahi, Mymensingh, Barishal, and Sylhet had higher proportions of individuals with low research literacy. However, it is important to note that the GIS application used in this study is exploratory and represents a novel methodological contribution; the lack of statistically significant regional findings indicates that spatial disparities should be interpreted cautiously and may sample limitations rather than true population differences. GIS and spatial analytics in this context are best viewed as hypothesis-generating rather than confirmatory tools, pointing to directions for further research. Furthermore, the findings suggest that research literacy levels do not significantly differ based on regions, emphasizing the need for special attention beyond divisional boundaries when addressing research literacy disparities.

In this study, machine learning techniques were used to predict research literacy levels. Both the Random Forest and CatBoost models demonstrated strong performance, achieving accuracy rates of 73.04% and 71.57%, respectively, with low log loss values of 0.57 and 0.56. Notably, while RF exhibited the highest precision value of 73.29%, CatBoost showed a commendable precision of 71.69%. Furthermore, RF attained the highest F1 score of 73.08%, with CatBoost closely following at 71.60%. These results are significant, considering that logarithmic loss rates below 2% are considered outstanding. Both CatBoost and RF models achieved maximum forecast accuracy with minimal log loss, outperforming other approaches comprehensively. Besides, in terms of AUC values, both CatBoost and RF exhibited superior discriminatory power, with scores of 0.79 and 0.78, respectively. Feature selection analysis indicated that participation in research courses outside the university was the most significant predictor, while the presence of researchers within the family had the least impact. This study’s findings highlight the effectiveness of CatBoost and RF models in predicting research literacy levels. The integration of machine learning approaches represents a methodological advance over traditional analyses, allowing robust prediction and ranking of risk factors by handling complex interactions among variables. These methods provide valuable tools for future research in identifying at-risk groups and guiding targeted educational or policy interventions, but their findings should be interpreted with caution in exploratory settings. There is no previous work that explicitly addresses research literacy, and this finding has limited comparison. In terms of information literacy, only a single work has utilized machine learning approaches for prediction. That is, the study involving 320 college students in China investigated information literacy learning and used supervised classification techniques including Decision Tree, K-Nearest Neighbors, Naive Bayes, Neural Networks, and Random Forest, found the Random Forest model performed the best out of all the algorithms examined, with 92.50% accuracy, 84.56% precision, 94.81% recall, 89.39% F1-score, and 0.859 Kappa coefficient^40^. These findings would like to highlight the potential of machine learning algorithms to advance literacy outcomes and inform educational interventions and prediction of their outcomes in diverse research literacy domains.

Based on the gaps and predictors identified in our study, we propose a series of targeted, evidence-based recommendations for universities, policymakers, and other stakeholders to enhance research literacy among students and graduates. Our analysis demonstrated that formal research courses, active research engagement, and mentorship were all associated with higher research literacy, while significant deficits were observed in areas such as awareness of predatory journals, open access, and research ethics. The following recommendations are grounded in these empirical findings and contextualized for practical implementation:

- Integrate Comprehensive Research Literacy Modules into Curricula: Our findings showed that participation in formal research courses—particularly those taken outside standard university programs—was the strongest predictor of research literacy. Universities should, therefore, make research literacy training a core curricular requirement at both undergraduate and postgraduate levels. These modules should provide hands-on instruction in skills such as identifying predatory journals, understanding peer review, navigating indexing databases, interpreting citation metrics, and upholding ethical standards, including recognizing and preventing plagiarism and research misconduct.
- Expand Access to High-Quality Workshops, Seminars, and Online Training: The significant impact of external research training in our study underscores the value of broadening opportunities for all students to attend workshops, webinars, and short courses, both on-campus and online. Such sessions, ideally offered in partnership with professional societies and external organizations, can expose students to current trends in publishing and research ethics, and reach those who may have less access to traditional university resources.
- Establish Structured Mentorship Programs: Our results demonstrated that active engagement in research and publication was linked to higher research literacy. Structured mentorship programs, pairing less-experienced students with research-active faculty or alumni, can provide practical guidance on research planning, publishing strategies, and ethical decision-making—empowering students to translate classroom learning into real-world research practices.
- Enhance Awareness of Emerging Scholarly Communication Practices: A notable proportion of participants in our study lacked awareness of newer research practices, such as preprints and open-access platforms. To close these gaps, institutions and national bodies should launch targeted awareness campaigns using digital tools, infographics, and e-learning modules, making this information accessible to all students—including those in under-resourced or remote settings.
- Establish Clear National and Institutional Policies on Research Literacy and Ethics: Our observation of widespread knowledge deficits, across demographic groups and regions, points to the need for national and institutional standards. Policymakers and university administrators should develop guidelines that set minimum research literacy competencies for graduation, clarify ethical publishing expectations, and establish clear reporting mechanisms for academic misconduct.
- Promote Inclusive and Equitable Interventions: Because our study found that research literacy gaps are not confined to specific regions or demographic groups, interventions must be designed for broad accessibility and equity. This could include targeted scholarships, outreach for underrepresented groups, and support programs to ensure that all students, regardless of background, can build strong research skills.
- Support Ongoing Faculty Development in Research Education: To sustain improvements in research literacy, faculty should be equipped with up-to-date knowledge on publishing, open science, and research ethics. Regular professional development and certification opportunities will enable instructors to serve as effective mentors and advocates for best practices in research training.

Strengths of this study include its comprehensive and systematic assessment of research literacy among university students and graduates. To our knowledge, this is the first study in Bangladesh—and among the first globally—to measure research literacy as a multi-dimensional construct encompassing key domains such as peer review, predatory publishing, open access, indexing, citation metrics, and research ethics. The study is methodologically innovative, combining traditional statistical analysis with GIS for spatial mapping and machine learning techniques for advanced predictive modeling and feature selection. The use of a structured, pilot-tested questionnaire with excellent internal consistency (Cronbach’s alpha = 0.939) further strengthens the credibility of the results. Together, these approaches enabled the identification of nuanced patterns, influential predictors, and region- or subgroup-specific disparities, offering actionable insights for educators, institutions, and policymakers.

Despite these strengths, several limitations should be acknowledged. The cross-sectional design restricts the ability to draw causal conclusions between the predictors and research literacy outcomes. The use of convenience and snowball sampling may have introduced sampling bias and limits the generalizability of the findings to the broader population of Bangladeshi students and graduates. The reliance on self-reported data may increase the risk of recall bias and social desirability effects, while the exclusive use of online surveys could exclude individuals with limited internet access or minimal connection with the research team. Although the research literacy scale demonstrated high reliability, formal construct validation and checks for item redundancy are needed in future research. While GIS and machine learning provided valuable new perspectives, their results should be interpreted with caution in light of sample and design limitations. Finally, as the choice of variables in the adjusted regression model was exploratory, the results should be interpreted with some caution. Future studies could address these limitations by employing longitudinal or experimental study designs, which would allow for causal inferences about the relationships between predictors and research literacy outcomes, as well as more diverse sampling strategies and continued refinement of measurement and analytic tools.

## Conclusions

In conclusion, this study offer the comprehensive and multi-dimensional assessment of research literacy and its predictors among university students and graduates in Bangladesh. The findings indicate that a significant proportion of participants exhibit poor research literacy, highlighting the urgent need for targeted interventions. Substantial deficits were identified in key domains such as awareness of predatory journals, research ethics, and open-access publishing, underscoring the importance of coordinated action. Based on our findings, we recommend that universities should integrate mandatory research literacy modules and practical skills training into undergraduate and postgraduate curricula. Expanding access to workshops, seminars, online courses, and formal mentorship programs will further support students’ research development. Higher education authorities and university administrators should establish national guidelines and minimum standards for research literacy, incentivize engagement in ethical research practices, and promote inclusive, equitable access to training and resources across all student groups. Additionally, targeted awareness campaigns and accessible digital resources are needed to address persistent knowledge gaps, particularly in emerging areas such as preprints and open science. Future research should prioritize the development and validation of robust research literacy assessment tools, the evaluation of intervention effectiveness, and the use of longitudinal and mixed-methods designs to monitor progress over time. By systematically embedding research literacy education and support into academic and policy frameworks, Bangladesh—and similar contexts—can foster a more ethical, capable, and globally competitive research culture, equipping graduates to navigate and contribute meaningfully to the evolving landscape of scientific publishing.

## Supplementary Information

Below is the link to the electronic supplementary material.

Supplementary Material 1

## Data Availability

The dataset will be made available to appropriate academic parties upon request from the corresponding author.
