# Supplementary material for: Research literacy and its predictors among university students and graduates identified by machine learning and spatial analysis
Source: Sci Rep. 2025 Oct 13;15:35622. doi: 10.1038/s41598-025-19488-4 (PMC12518792; doi:10.1038/s41598-025-19488-4)
Supplement: Supplementary file 1 — Supplementary Material 1 [file 41598_2025_19488_MOESM1_ESM.docx]

***Questionnaire:***

**Research literacy and its predictors among university students and graduates identified by machine learning and spatial analysis**

**Section 1: Socio-demographic Information**

- 1. Gender:

1. Male
2. Female
   1. Your Age:
   2. Relationship Status:
3. Married
4. Unmarried/ Single
5. In a relationship
6. Others
   1. What is the name of your district?
   2. What is your permanent residence?
7. Rural
8. Urban
   1. What’s your current living place?
9. With family
10. With friends
11. In dormitory
12. Other places
    1. What is your source of financial support?
13. Own
14. Family
15. Both
    1. What is your family's monthly income (only the income is mentioned here)?
    2. How much is your monthly income if you are employed yourself (only the income is mentioned here)?
    3. Do your family supportive to the research activities?
16. Yes
17. No
    1. Any of your family members involved with research activities?
18. Yes
19. No
    - 1. If yes, then what is the relationship with you?

**Section 2: Academic and research – training related information**

2.1. What is the name of your university for bachelor degree?

2.2. What is the name of your bachelor's department?

2.3. What is your bachelor's session?

2.4. What is your last published bachelor's result?

2.5. Are your bachelor's and master's from the same university?

1. Yes
2. No

2.5.1. If no, what is your master’s university name?

2.6. What is the name of your master's department?

2.7. What is your master's session?

2.8. What is your last published master's result?

2.9. Have you been in any sessions jam in academic education?

1. Yes
2. No

2.10. Were there any research courses in your bachelor's curriculum?

1. Yes
2. No

2.11. Were there any research courses in your master's curriculum?

1. Yes
2. No

2.12. Have you understood the research methodology easily in the research course from the bachelor's and master's curriculum that has helped or will help your thesis or publication?

1. Not at all
2. Somewhat
3. Moderate
4. Highly

2.13. Have you taken research courses from any institutions outside the university education?

1. Yes
2. No

2.13.1. If yes, then how many research courses?

2.14. Have you understood the research methodology easily in the research course outside the university education that has helped or will help your thesis or publication?

1. Not at all
2. Somewhat
3. Moderate
4. Highly

**Section 3: Research publication and career – related information**

3.1. Do you have any plan to take research as a career?

1. Yes
2. No

3.1.1. If Yes, what are the motivations/reasons to choose this one as your career goal? (Multiple choice question)

 Interest in academic job or university faculty

 Interest in higher study

 Working at development sector, NGOs

 Building career as a researcher

 Working at research and development sector

 Gaining professional recognition

 Requirement of institution or program

 Gaining personal recognition

 Interest in interdisciplinary collaboration and networking with peers

 Developing research skills

3.2. Had you involved with any research work other than thesis?

1. Yes
2. No

3.2.1. If yes, What is the current status of your other research work?

1. Continuing work, will submit in journal
2. Submitted to journal, under-review
3. Published at least a paper

3.3. What is your total number of published research papers?

3.4. Are you currently involved in a research related field?

1. No research – related job
2. Work alone
3. Working with a thesis supervisor or someone else
4. Working in a research institute (paid)
5. Working in a research institute (non - paid)
6. Working through several of the above-mentioned options

4.5. Have you finished your thesis or are you working on the thesis or will not do the thesis?

1. Finishing my thesis
2. Have started my thesis, but it's not over yet
3. Will not: There are necessary results for the thesis, but there is no desire
4. Still studying undergrad, will think about taking a thesis when get to the master's
5. There is no opportunity to do a thesis (will not get a thesis on the basis of results, or there is no thesis in the institute)

**Section 4: Research Literacy**

**4.1. Peer-Review Process:** To what extent do you know the details of the peer-review process within academic publishing, including the nuanced roles undertaken by reviewers, and editors, and the comprehensive evaluation criteria applied?

1. Not at all familiar
2. Slightly familiar
3. Moderately familiar
4. Very familiar

**4.2. Predatory Journals:** How confident are you in your ability to effectively distinguish between publications emanating from predatory journals and those originating from reputable and esteemed sources?

1. Not at all confident
2. Slightly confident
3. Moderately confident
4. Very confident

**4.3. Open Access Publishing:** To what degree do you possess a comprehensive understanding of various open access publishing models, encompassing hybrid approaches and subscription-based options?

1. Not at all familiar
2. Slightly familiar
3. Moderately familiar
4. Very familiar

**4.4. Indexing Sites:** How familiar are you with prominent indexing sites such as Scopus, PubMed, Web of Science, Google Scholar, and DOAJ, which play pivotal roles in the academic literature Domain?

1. Not at all familiar
2. Slightly familiar
3. Moderately familiar
4. Very familiar

**4.5. Citation Metrics:** To what extent are you familiar with key citation metrics, such as citation count, i-10 index, and h-index, which play crucial roles in evaluating the impact and influence of

scholarly publications?

1. Not at all familiar
2. Slightly familiar
3. Moderately familiar
4. Very familiar

**4.6. Impact Factor or Cite Score:** Can you articulate your understanding of journal Impact Factor or Cite Score, metrics that hold significance in assessing the influence and reach of academic journals?

1. Not at all understanding
2. Slightly understanding
3. Moderately understanding
4. Very understanding

**4.7. Directory of Open Access Journals:** Are you acquainted with the Directory of Open Access Journals (DOAJ), a repository that indexes high-quality, open-access, peer-reviewed journals?

1. Not at all familiar
2. Slightly familiar
3. Moderately familiar
4. Very familiar

**4.8. Awareness of Beall’s List**: Are you familiar with Beall’s List, a compilation identifying potentially predatory publishers and journals within the academic realm?

1. Not at all familiar
2. Slightly familiar
3. Moderately familiar
4. Very familiar

**4.9. Plagiarism Awareness:** How well can you elucidate your understanding of the concept of data fabrication and plagiarism and their far-reaching implications within the academic context?

1. Not at all understanding
2. Slightly understanding
3. Moderately understanding
4. Very understanding

**4.10. Preprint Awareness:** To what extent are you acquainted with the concept of preprints in

academic publishing, which involves the dissemination of early versions of research papers before formal peer review?

1. Not at all understanding
2. Slightly understanding
3. Moderately understanding
4. Very understanding
